# Multiomics analysis revealed miRNAs as potential regulators of the immune response in *Carassius auratus* gills to *Aeromonas hydrophila* infection

**DOI:** 10.3389/fimmu.2023.1098455

**Published:** 2023-02-03

**Authors:** Jiaxin Huo, Xiucai Hu, Jie Bai, Aijun Lv

**Affiliations:** Tianjin Key Lab of Aqua-Ecology and Aquaculture, College of Fisheries, Tianjin Agricultural University, Tianjin, China

**Keywords:** *Carassius auratus*, miRNA, iTRAQ, immune response, histopathology, gills

## Abstract

The gill of fish is an important immune organ for pathogen defense, but its microRNA (miRNA) expression and regulatory mechanism remain unclear. In this study, we report on the histopathological and immunohistochemical features of the gills of the crucian carp *Carassius auratus* challenged with *Aeromonas hydrophila*. Small RNA libraries of the gills were constructed and sequenced on the Illumina HiSeq 2000 platform. A total of 1,165 differentially expressed miRNAs (DEMs) were identified in gills, of which 539 known and 7 unknown DEMs were significantly screened (*p* < 0.05). Gene Ontology (GO) and Kyoto Encyclopedia of Genes and Genomes (KEGG) enrichment analyses revealed that the potential target genes/proteins were primarily involved in 33 immune-related pathways, in which the inflammatory responses were focused on the Toll-like receptor (TLR), mitogen-activated protein kinase (MAPK), and nuclear factor kappa B (NF-κB) signaling pathways. Moreover, the expression levels of 14 key miRNAs (e.g., miR-10, miR-17, miR-26a, miR-144, miR-145, and miR-146a) and their target genes (e.g., *TNFα*, *TLR4*, *NF-κB*, *TAB1*, *PI3K*, and *IRAK1*) were verified. In addition, the protein levels based on isobaric tags for relative and absolute quantification (iTRAQ) were significantly associated with the results of the quantitative real-time PCR (qRT-PCR) analysis (*p* < 0.01). miR-17/pre-miR-17 were identified in the regulation expression of the NF-κB target gene, and the phylogenetic tree analysis showed that the pre-miR-17 of *C. auratus* with the closest similarity to the zebrafish *Danio rerio* is highly conserved in teleosts. This is the first report of the multi-omics analysis of the miRNAs and proteins in the gills of *C. auratus* infected with *A. hydrophila*, thus enriching knowledge on the regulation mechanism of the local immune response in Cyprinidae fish.

## Highlights

Multi-omics analysis of the miRNAs and iTRAQ profiles was first performed in the gills of *C. auratus* upon *A. hydrophila* infection.

The miRNAs (e.g., miR-17, miR-26a, miR-144, and miR-146a) play crucial roles in the gill local immune response of *C. auratus* to bacterial infection.

The target genes of miRNAs are involved in signaling pathways such as TLR, MAPK, and NF-κB inflammatory responses.

## Introduction

MicroRNAs (miRNAs) are small endogenous non-coding RNAs with a length of approximately 20–24 bases that can bind to the 3′ untranslated region (3′-UTR) of messenger RNAs (mRNAs), thereby guiding the degradation of the target genes or inhibiting translation to negatively regulate the gene expression (1, 2). Since their first discovery in *Caenorhabditis elegans*, miRNAs have encompassed cellular functions in diverse biological processes, such as cell growth, proliferation and differentiation, development, immunity, and apoptosis (3, 4). In the past decades, some studies combined molecular immunity to prove that miRNAs are pivotal regulators of the inflammatory response (5–7). Furthermore, high-throughput sequencing has been widely used for miRNA expression profiling analysis in fish (3, 8). The innate immune system involves different signaling pathways that are regulated by complex mechanisms, most of the miRNAs of which are still unclear in fish (9, 10). In teleosts, recent studies have suggested that miR-21 targets *IRAK4* to inhibit excessive immune response (11), that miR-146 shows immune-related inducible expression in the zebrafish *Danio rerio* after *Aeromonas hydrophila* infection (12), and that miR-144 is differentially expressed to modulate the host response in the bacteria-infected miiuy croaker *Miichthys miiuy* (13). In addition, several miRNAs (e.g., miR-122, miR-192, and miR-148) have been identified to regulate the target genes that participate in innate immunity (14–16). Accumulating evidence demonstrated that, in the immune response process against bacterial infection, most miRNAs focus on regulating the gene expression in the Toll-like receptors (TLRs), C-type lectin receptors (CLR, mitogen-activated protein kinases (MAPKs) and MyD88-mediated nuclear factor kappa B (NF-κB) signaling pathways (4, 13, 16, 17). Recently, some miRNAs have been found to be involved in the regulation of signal transduction, growth, and immunity in the crucian carp *Carassius auratus* (5, 18). However, the regulation mechanisms of miRNA-mediated bacterial infection are still poorly studied in teleosts.

As a primary immune organ, the gill is considered a large mucosal surface with a variety of functions for respiration, osmotic regulation, and toxicological response (19). Recent studies have reported the gene expression patterns of gills after infection and investigated their immune response at different molecular levels (20, 21), in which immune-related genes (e.g., *TLR4*, *IRAK1*, *TNF*, and *IL-1β*) were found to be differentially expressed in the gills of teleosts (22–24). Notably, the miRNA (e.g., miR-135b, miR-146, and miR-10a-5p) expression in the gills of the Atlantic killifish *Fundulus heteroclitus* and the grass carp *Ctenopharyngodon idella* infected with *A. hydrophila*, as well as the zebrafish *D. rerio* after *Staphylococcus aureus* infection, has verified their roles in the gene regulatory network (20, 25, 26). Although previous studies have shown that gills play important roles in the immune system as the first line of defense against the invasion of pathogens, the mechanisms of the gill immune response of the miRNAs and target genes regulating bacterial infection remain unclear in fish.

The crucian carp *C. auratus* is a major freshwater species cultured for human consumption in China (5, 18). Nevertheless, the outbreak of bacterial diseases of *C. auratus* is has become a serious problem, causing economic losses (8, 18, 27). As an important Gram-negative opportunistic pathogen, *A. hydrophila* is widely distributed in the water environment (27). Recently, the characterization of the miRNAs and target genes in the internal organs (e.g., kidney, spleen, and liver) and skin of *C. auratus* has been performed using deep sequencing analysis (5, 18). Moreover, proteomic analyses (e.g., iTRAQ and 2-DE/MS) were performed in zebrafish gills and crucian carp skin after *A. hydrophila* infection (19, 28). However, the miRNA expression and its regulatory mechanism in the gills of *C. auratus* are still unclear. The present study used conventional histopathological examination, multi-omics analysis of the mRNA and protein profiles, and the high-throughput small RNA (sRNA) transcriptome sequencing technique to construct an sRNA library of the gills of *C. auratus* infected with *A. hydrophila* based on the Illumina HiSeq 2000 platform. The results of this study provide a scientific basis for elucidating the miRNAs and target genes involved in the molecular mechanisms of the gill immune response of teleost fish.

## Materials and methods

### Sample and challenge

*C. auratus* (average weight, 50 g) were obtained from Tianshi Fisheries Development Co., Ltd., Tianjin, China. Prior to the bacterial infection, the fish were acclimatized at a temperature of approximately 25°C in freshwater tanks (45 L) for 2 weeks and fed commercial dried pellets (Tongwei Co., Ltd., Chengdu, China) twice daily. In the infection experiment, the fish were immersed in *A. hydrophila* culture with a final concentration of 1 × 10^8^ cfu/ml and then quickly transferred into freshwater after immersion for 3 h, in accordance with a previous report by Wang et al. (27). A total of 60 control and infected fish at 0, 6, and 12 h were randomly selected and aseptically excised for the collection of gill tissue samples. Three mixed gill samples were immediately frozen in liquid nitrogen and stored in a refrigerator at −80°C for later extraction of RNA. The gill samples in the control and infected groups were labeled mGC and mGT, respectively.

### Pathology, immunohistochemistry, and phagocytic activity analysis

Fish were randomly selected and dissected for histopathological examination. After deep anesthesia with MS-222 (200 mg/l), the mucosal (i.e., gill, skin, and intestine) and visceral (i.e., liver, kidney, and spleen) tissue samples were fixed in 10% neutral buffered formalin for at least 24 h, then dehydrated using ascending concentrations of 70%–100% ethanol, cleared in xylene, and finally embedded in paraffin wax. Tissue sections of 5-μm thickness were cut on a microtome (Leica RM 2125, Wetzlar, Germany), stained with hematoxylin and eosin (H&E), and examined using a light microscope (Leica DM 5000, Wetzlar, Germany). For the preparation of the rabbit polyclonal antibody, inactivated *A. hydrophila* bacteria were used as antigens to immune rabbits. The antigen was prepared using equal amounts of Freund’s adjuvant (Sigma, St. Louis, MO, USA) according to the manufacturer’s guidelines. Two rabbits were immunized with five hypodermic injections. Three days after the fifth injection, the rabbits were bled and the antiserum was detected and stored at −80°C. Immunohistochemical (IHC) staining experiments were performed using a Solarbio kit (Beijing, China) following the manufacturer’s protocols. Phagocytosis assays were performed as described by Santos et al. (29). Briefly, 0.5 ml of blood was drawn by caudal puncture using a disposable syringe with a 0.7 × 25-mm needle, slightly moistened with diluted heparin solution (5,000 IU heparin in 50 ml of saline, 0.7%), and then immediately utilized for determination of the phagocytic activity.

### Total RNA extraction

Gill samples were extracted using the RNAeasy™ Animal RNA Isolation Kit with Spin Column (code no. R0026; Beyotime, Shanghai, China) according to the manufacturer’s instructions. The NanoPhotometer^®^ spectrophotometer (NanoDrop, Wilmington, DE, USA) and agarose gel electrophoresis were utilized to check the purity of the RNA. The integrity and quality of RNA for the construction of the sRNA libraries were examined using the Agilent Bioanalyzer 2100 system (Agilent Technologies, Santa Clara, CA, USA) to ensure that high-quality samples were used for sequencing.

### Illumina sequencing

According to the conventional method (29), a total of 3 μg of total RNA per sample was used as the input material for the RNA sample preparations. Two sRNA libraries (i.e., mGC and mGT) were constructed from the gill samples of the control group and the infected fish group. The NEBNext^®^ Multiplex Small RNA Library Prep Set for Illumina^®^ (NEB, Ipswich, MA, USA) was used to generate a sequencing library, and the TruSeq SR Cluster Kit v3-CBOT-HS (Illumina, San Diego, CA, USA) was utilized to execute the index code samples on the CBOT Cluster generation system. After clustering, the library was sequenced on the Illumina HiSeq 2000 platform, and a 50-bp single-ended read was produced by BGI Genomics Co., Ltd. (Shenzhen, China).

### Analysis of the sequencing data and annotation of sRNAs

According to the recent report by Bai et al. (18), after completion of the high-throughput sRNA sequencing, overlapping and contaminated sequences and low-quality reads from the original data were removed and the length distribution of the sRNAs was determined. Non-coding RNAs such as ribosomal RNA (rRNA), transfer RNA (tRNA), and small nuclear RNA (snRNA) were identified by comparing with the Rfam database and GenBank, and the known miRNAs in the sample were identified by comparing with the miRNAs in the specified range in miRBase. sRNAs related to the repeat sequence were identified by alignment with the repeat sequence, and the mRNA degradation fragments were identified by comparison with the exons and introns. Moreover, the sRNAs were classified and annotated according to priority, and MIREAP was used to predict novel miRNAs and to determine the step ring or hairpin structure.

### Differential expression and phylogenetic analysis of miRNAs

To analyze the differential expression of the miRNAs, their expression levels were standardized to calculate the expression in transcripts per million (TPM) for each library, as described by He et al. (7). The expression levels of the miRNAs in the control and infected samples (mGC/mGT) were determined with the DEGseq program. The Benjamini–Hochberg method was used for multiple verification and correction. By default, a correction *p*-value <0.05 was set as the threshold for significant differential expression.

The pre-miR-17 sequence of the crucian carp *C. auratus* was obtained using high-throughput sequencing. The sequences of 26 different species from the miRBase database were selected for molecular identification analysis, including the annotations, corresponding species names, and precursor sequences (**Supplementary Table S1**). MEGA 7.0 software was used for multiple sequence alignment of pre-miR-17, and maximum likelihood estimation (MLE) was utilized for the pre-miR-17 phylogenetic tree (bootstrap = 1,000).

### Protein extraction, iTRAQ, and screening of DEPs

Protein extraction of the gill samples and isobaric tags for relative and absolute quantification (iTRAQ) labeling were performed as previously described (19). The protein concentration was determined using a BCA protein assay kit (Sangon Biotech, Shanghai, China), and the proteins were visualized with SDS-PAGE to determine their quality. In the iTRAQ analysis, protein identification and quantification were performed at Genomics Co., Ltd. (Shenzhen, China). For protein quantitation, those with ratios with *p* < 0.05 and fold change >1.2 were considered significant differentially expressed proteins (DEPs).

### GO and KEGG enrichment analyses

Gene Ontology (GO) and Kyoto Encyclopedia of Genes and Genomes (KEGG) functional enrichment analyses were performed for the miRNA candidate target genes with differential expression. The commonly used prediction algorithms miRanda, pITA, and RNA hybridization were used to predict the potential miRNA targets (18).

### qRT-PCR analysis of the miRNAs and target genes

The miRNAs were extracted using the RNAeasy™ Animal Small RNA Extraction Kit (code no. R0028; Beyotime, Shanghai, China), and the Mir-X miRNA First-Strand Synthesis Kit (code no. 638315; Takara, Dalian, China) was used to synthesize the complementary DNAs (cDNAs). The candidate miRNAs were selected from the differentially expressed miRNAs (DEMs), and the cDNAs of the fish in the control and infected groups were selected as templates, with each sample containing three repeats. Quantitative real-time PCR (qRT-PCR) reaction was carried out with tail primers and universal primers and with 5.8S rRNA as the internal reference gene, verifying the expression of the DEMs. According to the results of the qRT-PCR and RNA-seq, changes in the expression of the miRNAs after infection were analyzed. **Supplementary Table S2** lists the primer sequences used for the qRT-PCR analysis. In addition, the RNAhybrid software was used to predict the potential target genes. The experiment was repeated with the previously obtained RNA samples. Furthermore, using *β-actin* as the internal reference gene, the spatiotemporal expression was determined for the immune-related genes in both control and infected fish.

## Results

### Pathological and immunohistochemical features of the gills of *C. auratus* infected with *A. hydrophila* and hemocyte phagocytic activity

**Figure 1:**
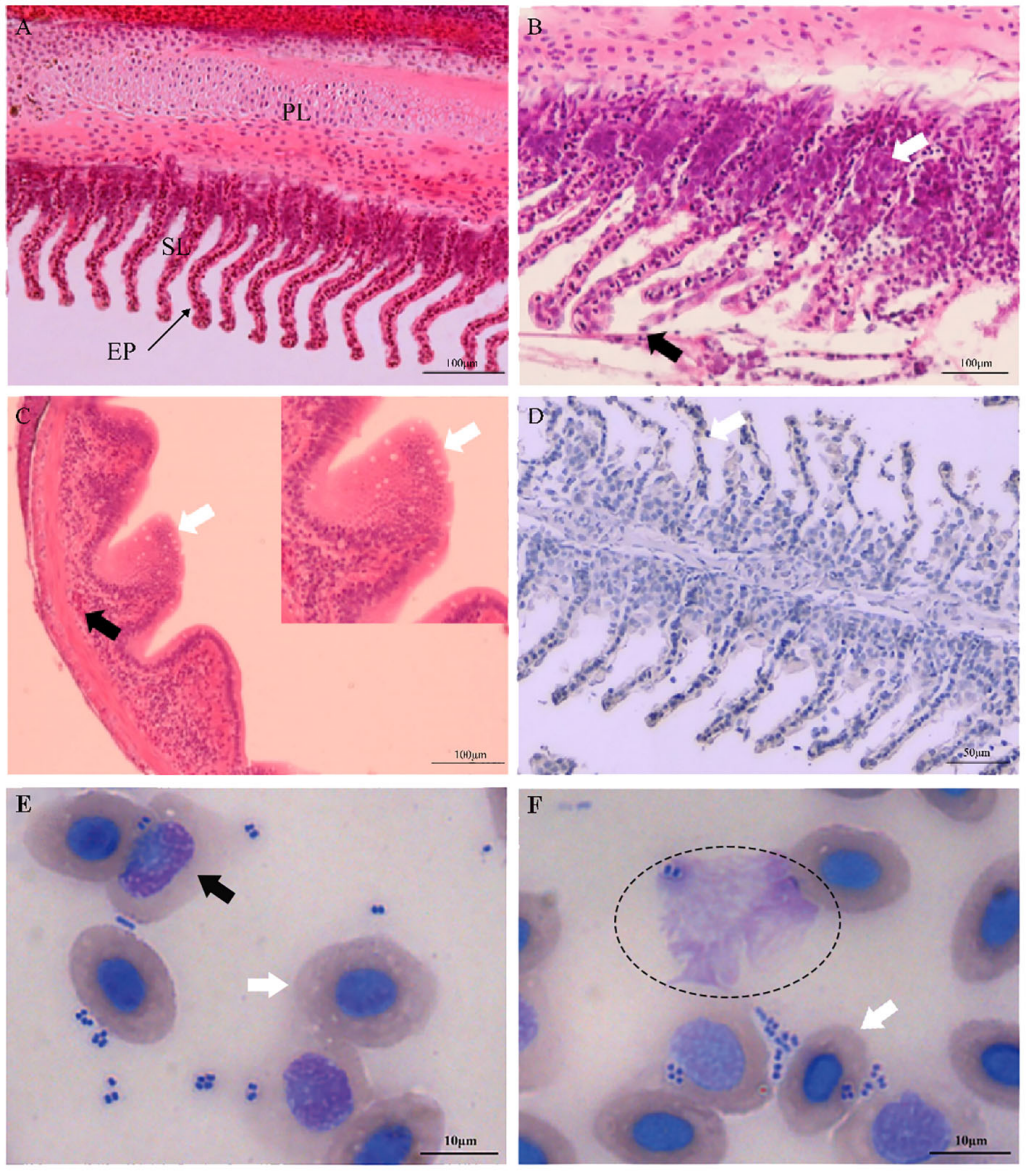
Histopathological changes and hemocyte phagocytosis of *Carassius auratus* infected with *Aeromonas hydrophila*. **(A)** Gills of the unexposed control fish. *PL*, primary lamellae; *SL*, secondary lamellae; *EP*, epithelial cell. **(B)** Exfoliation and necrosis of gill respiratory epithelial cells (*black arrow*) and extensive inflammatory cell infiltration (*white arrow*). **(C)** Intestinal villus atrophy, dehydration, vacuolization, and necrosis (*white arrow*), and lamina propria submucosa hemorrhage (*black arrow*). **(D)** Immunohistochemical staining of the gill tissues of *A hydrophila*, with positive staining appearing brown (*white arrow*). **(E, F)** Hemocyte phagocytosis of *C auratus* infected with *A hydrophila*. Phagocytic activity of *A hydrophila* is displayed by neutrophils (*black arrow*), macrophages (*elliptic circle*), and blood cells (*white arrow*).

Clinically diseased *C. auratus* infected with *A. hydrophila* exhibited hyperemia, hemorrhage, and inflammatory lesion of the mucosal tissues in the gills, skin, and intestine, as well as congestion of the visceral organs. Compared with the gills of control fish, histopathological examination of the infected fish revealed high inflammatory cell infiltration in the base of the secondary gill lamellae, and the respiratory epithelial cells of the secondary lamellae showed hyperplasia, sloughing, and necrosis (**Figures 1A, B**). Severely damaged intestinal villi, atrophic change, and extensive vacuolization with hemorrhage in the intestinal mucosa were also detected (**Figure 1C**). In addition, the primary manifestations on the skin were hyperplasia of the epidermal mucus cells with hemorrhagic lesions, and different degrees of pathological changes such as hyperemia, hemorrhage, and inflammatory cell infiltration that occurred in the liver, kidney, and spleen. Immunohistochemical staining in the gills also revealed positive signals that were mainly distributed in the secondary gill epithelial cells (**Figure 1D**). In the peripheral blood of *C. auratus*, different types of leukocytes and hemocytes could phagocytize bacteria *in vitro*. Most of the *A. hydrophila* were observed to adhere to the surface of hemocytes and leukocytes, and some bacterial cells were phagocytized in hemocytes, particularly with the macrophages and neutrophils producing pseudopodia or forming emboli (**Figures 1E, F**).

### Summary of the miRNA transcriptome in the gills of *C. auratus*

**Figure 2:**
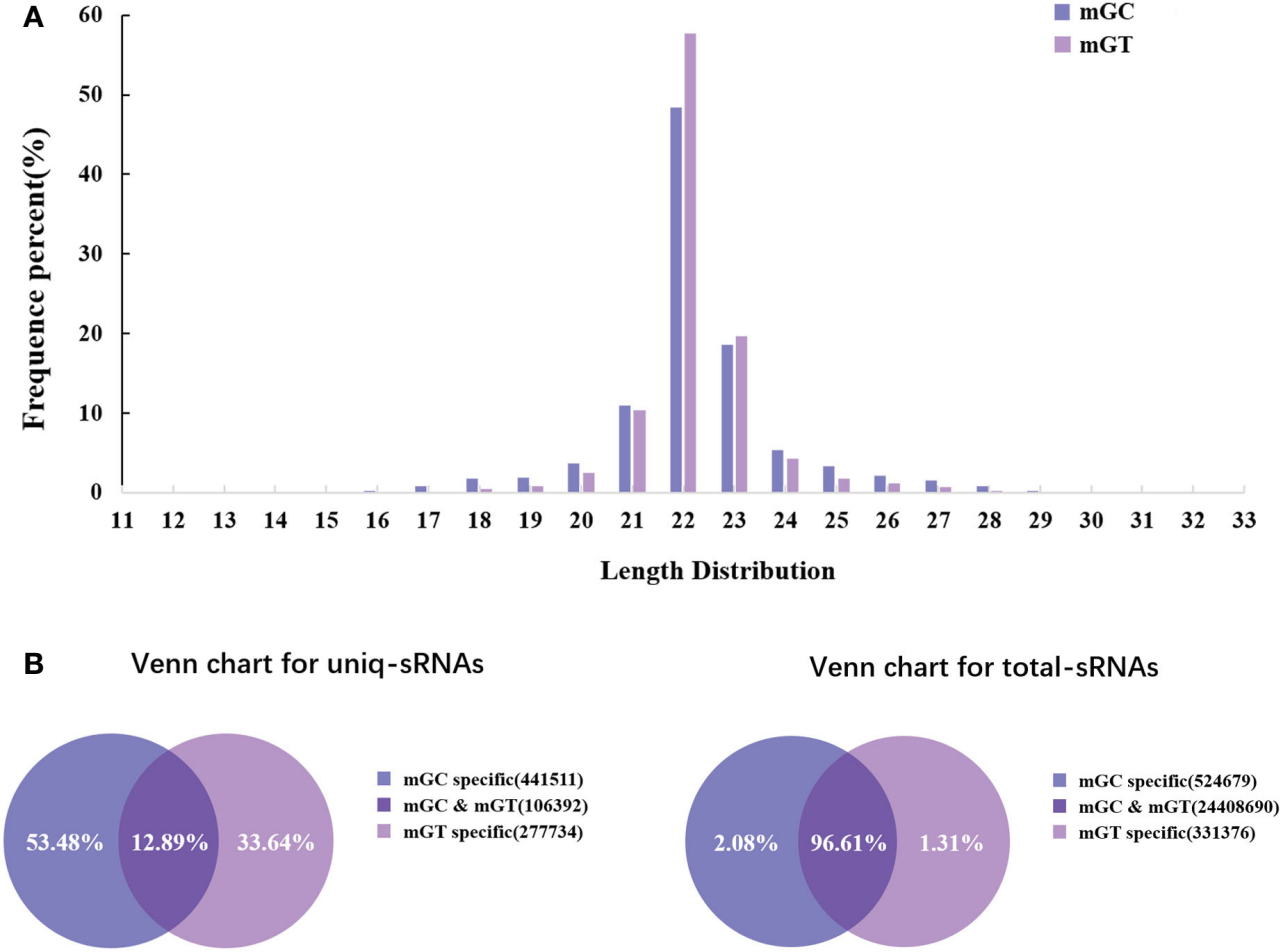
Overview of the small RNA (sRNA) sequencing libraries of the gills. **(A)** Length distribution of the sRNAs from control and infected *Carassius auratus*. **(B)** Common and specific sRNA sequences in the two libraries. Unique sRNAs (*left*) and total sRNAs (*right*).

In order to investigate the miRNAs related to bacterial infection and mucosal immune response, the sRNA libraries from the gills of *C. auratus* in the control and infected groups were constructed using the Illumina HiSeq 2000 platform. The results showed that a total of 25,628,924 original reads were generated, with 12,411,378 (98.13% of raw reads) and 12,853,367 (99.71% of raw reads) clean reads obtained from the control and infected gills, respectively (**Supplementary Table S3**). To further assess the changes in the sRNAs of *C. auratus*, we examined the length distribution of all sRNA reading fragments in the two libraries (mGC and mGT). Most of the unique sRNAs from the gills were between 20 and 24 nt, with a peak distribution of 22 nt, which is consistent with the typical size of products processed using Dicer (**Figure 2A**).

**Figure 3:**
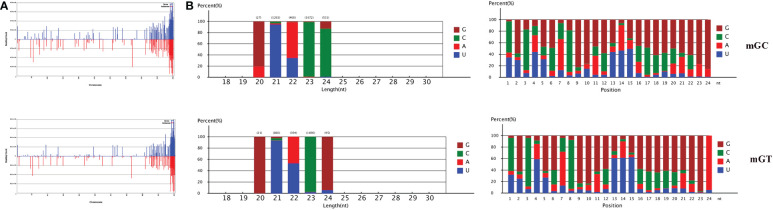
Distribution of the microRNAs (miRNAs) in *Carassius auratus* gills. **(A)** Fragments on each chromosome. **(B)** Analysis of first nucleotide bias (*left*) and position nucleotide bias (*right*).

In addition, 547,903 and 384,126 sRNA sequences were obtained in the control and infected gills, respectively. Of these, 65,706 (11.99%) and 47,614 (12.4%) were identified as miRNAs in the gills. The remaining sequences comprised other types of RNAs, including rRNA, snRNA, small nucleolar RNA (snoRNA), and tRNA (**Table 1**). To examine the overall consistency of the sequencing data of the miRNAs, the unique and total distributions of the common and unique sequences in the mGT and mGC libraries were calculated (**Figure 2B**). The proportion of common sequences was 106,392 (12.89%), and the total common sequences accounted for 24,408,690 (96.61%) of the total clean reads. To establish the sRNA genome map of *C. auratus*, their expression and distribution in the genome were predicted, and then the miRNAs were compared to the corresponding precursors of *C. auratus* and *D. rerio* species in miRBase constructed to obtain the repertoire of the miRNAs in the gills. The results showed that 9,210,212 (74.21%) and 10,144,917 (78.93%) sRNA bands and 57,972 (10.58%) and 41,768 (10.87%) specific sRNAs were located in the genome of *C. auratus* (**Figure 3A**). In mGC and mGT, analysis of the first nucleotide bias showed that the content of cytosine (C) was dominant in all known miRNA sites, with a proportion of more than 50% (**Figure 3B**, left), while nucleotide bias analysis of the known miRNA locations showed that guanine (G) content was dominant in the gills (**Figure 3B**, right).

**Table 1:** Categorization of the non-coding and organellar small RNAs in the gills of *Carassius auratus*.

Type of sRNA	mGC		mGT	
	No. of reads	Percentage	No. of reads	Percentage
miRNA	65,706	11.99	47,614	12.4
rRNA	32,334	5.9	28,211	7.34
snRNA	2,508	0.46	1,736	0.45
snoRNA	963	0.18	614	0.16
tRNA	8,944	1.63	7,212	1.88
Unannotated	412,968	75.37	281,067	73.17
Exon: antisense	3,018	0.55	1,569	0.41
Exon: sense	19,268	3.52	14,908	3.88
Intron: antisense	967	0.18	514	0.13
Intron: sense	1,227	0.22	681	0.18
Total	547,903	100	384,126	100

miRNA, microRNA; rRNA, ribosomal RNA; snRNA, small nuclear RNA; snoRNA, small nucleolar RNA; tRNA, transfer RNA.

### Differential expression profiling of the miRNAs and screening and identification of the key DEMs in the gills of *C. auratus*

**Figure 4:**
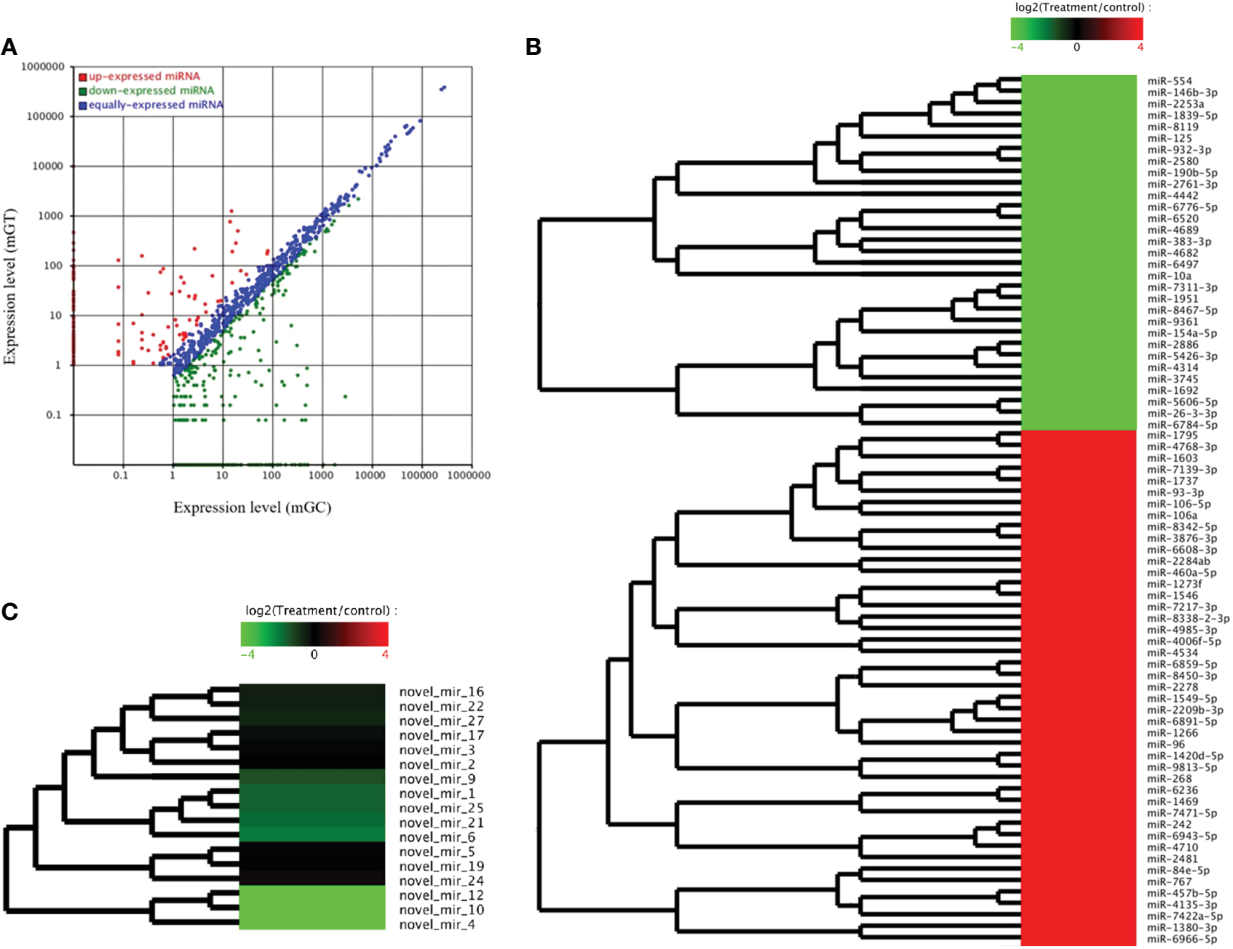
Analysis of the differentially expressed miRNAs (DEMs) in the gills of *Carassius auratus* infected with *Aeromonas hydrophila*. **(A)** Scatter plots of the DEMs. *Each point* represents one miRNA, and the *abscissa* and *ordinate values* indicate the expression levels of the miRNAs in the control and infected fish, respectively. **(B, C)** Cluster analysis of the partially known miRNAs **(B)** and the novel miRNAs **(C)** with similar expression patterns.

**Figure 5:**
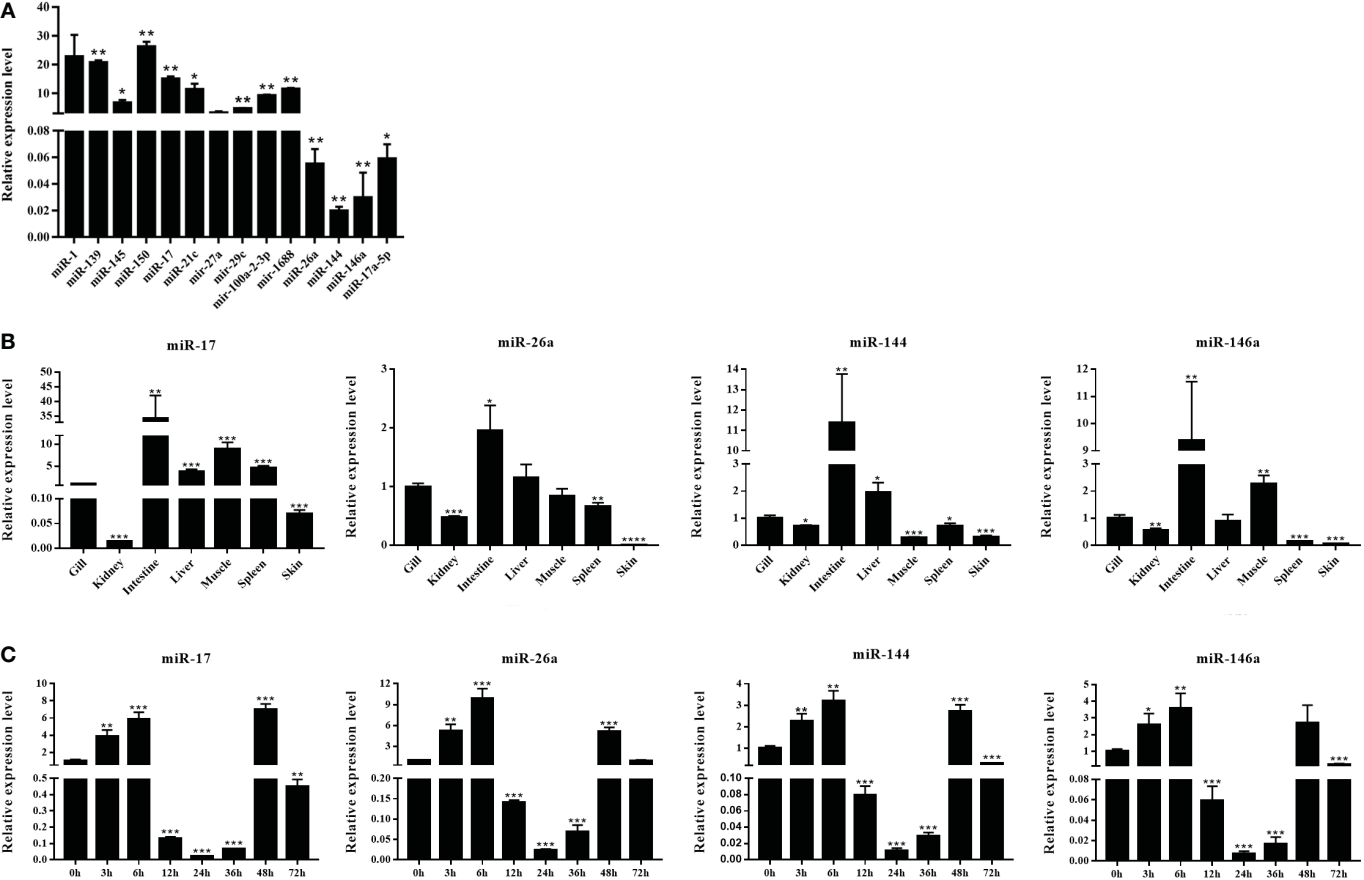
Expression analysis of the differentially expressed miRNAs (DEMs) in normal and *Aeromonas hydrophila*-infected *Carassius auratus*. **(A)** Validation of the relative expression using quantitative real-time PCR (qRT-PCR) of the 14 DEMs in the gills. **(B)** Tissue distribution of four DEMs—miR-17, miR-144, miR-146a, and miR-26a—in healthy *C auratus*. **(C)** Temporal expression of the four key miRNAs in infected gill tissues. All data were from three independent triplicate experiments (*p < 0.05, **p < 0.01). ***P<0.001, ****P<0.0001.

The DEMs were screened for *C. auratus* following *A. hydrophila* infection and the results revealed the differential expression profiles of 1,165 DEMs associated with the gill immune response of *C. auratus* (**Figure 4A**). Of these, 539 DEMs screened from 1,148 known DEMs were observed as significant (*p* < 0.05). Cluster analysis was performed on both known and novel miRNAs that showed similar expression patterns, which indicated that the expression of several known miRNAs (e.g., miR-10a, miR-26-3-3p, miR-125, miR-146b-3p, miR-154a-5p, miR-190b-5p, miR-383-3p, miR-554, and miR-932-3p) was significantly downregulated (**Figure 4B**). In addition, 17 unknown miRNAs were obtained after infection, of which seven representative novel miRNAs (i.e., novel-miR-1, novel-miR-4, novel-miR-6, novel-miR-10, novel-miR-12, novel-miR-21, and novel-miR-25) showed significant differential expression out of all those downregulated in gills (*p* < 0.05) (**Figure 4C**). To further verify the results of the transcriptome analysis, we selected 14 key DEMs for detection in the qRT-PCR assay, and the changes in their relative expression were compared with those of the RNA-seq expression profile analysis. The results showed that the expression levels of miR-1, miR-27a, miR-145, miR-146a, miR-150, and miR-100a-2-3p were upregulated, while those of miR-10, miR-17, miR-21, and miR-26a were downregulated, which is consistent with the regulation direction of the RNA-seq expression (**Figure 5A**). On the other hand, the spatiotemporal expression patterns of miR-17, miR-26a, miR-144, and miR-146a in the gills were further examined, and the results showed that these four miRNAs were significantly expressed in all detected tissues, such as the gill, liver, spleen, and muscle, with particularly high expression in the intestine (**Figure 5B**). The “up–down–up” trend in expression was observed in the infected gills at the early [0–6 hours post-infection (hpi)], middle (12–36 hpi), and late (48–72 hpi) stages, with the expression levels being concurrently highest at 6 and 48 hpi (**Figure 5C**). The differential expression of the selected 20 known DEMs, including miR-10, miR-21, miR-100, miR-145, miR-148, miR-192, and miR-27b-3p, was significantly detected after *A. hydrophila* infection, indicating that these miRNAs were involved in the gill immune response process. The significant differential expression levels of the representative DEMs are shown in **Table 2** (**Supplementary Table S4**).

**Table 2:** Differential expression of the known and unknown miRNAs in the gills of *Carassius auratus* challenged with *Aeromonas hydrophila*.

miR_name	Fold change	*p*-value	Species	Target gene	Reference
miR-10	−6.05	6.75e−37	Sea cucumber (*Apostichopus japonicus*)	*TBC1D5*	30
miR-21	−0.20	0	Miiuy croaker (*Miichthys miiuy*)	*IRAK4*	11
miR-27b-3p	−0.98	5.28e−134	Common carp (*Cyprinus carpio*)	*CYP1B1*	31
miR-29c	−0.34	1.43e−04	Mouse (*Mus musculus*); human (*Homo sapiens*)	*RAG1*	32
miR-100	0.12	1.44e−06	Human (*Homo sapiens*)	*ATM*	33
miR-128	−0.31	2.67e−174	Miiuy croaker (*Miichthys miiuy*)	*TAB2*	34
miR-132-2p	−0.92	1.84e−22	Mouse (*Mus musculus*)	*SIRT1*	35
miR-142a-3p	−0.89	9.04e−28	Grass carp (*Ctenopharyngodon idella*)	*TLR5*	36
miR-145	1.14	8.35e−40	Human (*Homo sapiens*)	*PAK4*	37
miR-145-5p	0.14	0.004	Miiuy croaker (*Miichthys miiuy*)	*MDA5*	38
miR-148	−0.27	4.19e−28	Miiuy croaker (*Miichthys miiuy*)	*MyD88*	16
miR-150	0.10	4.57e−09	Japanese flounder (*Paralichthys olivaceus*)	*LMP2L*	39
miR-155	−0.85	1.13e−17	Channel catfish (*Ictalurus punctatus*)	*SOCS1*	40
miR-200a	−0.57	2.12e−160	Wuchang bream (*Megalobrama amblycephala*)	*MAPK1*	41
miR-206	−0.23	0	Human (*Homo sapiens*)	*FAIM*	42
miR-210	−0.98	1.35e−65	Miiuy croaker (*Miichthys miiuy*)	*RIPK2*	43
miR-17	−13.28	0	Crucian carp (*C. auratus*)	*NF-κB*	This study
miR-26a	−0.14	7.78e−21	Crucian carp (*C. auratus*)	*TLR4*	This study
miR-144	0.16	1.11e−05	Crucian carp (*C. auratus*)	*TAB1*	This study
miR-146a	0.13	6.15e−199	Crucian carp (*C. auratus*)	*IRAK1*	This study
novel-miR-1	−1.53	3.96e−06	Crucian carp (*C. auratus*)	Unknown	This study
novel-miR-4	−9.52	7.96e−29	Crucian carp (*C. auratus*)	Unknown	This study
novel-miR-6	−1.92	0.002	Crucian carp (*C. auratus*)	Unknown	This study
novel-miR-10	−7.60	3.83e−08	Crucian carp (*C. auratus*)	Unknown	This study
novel-miR-12	−6.92	2.30e−05	Crucian carp (*C. auratus*)	Unknown	This study
novel-miR-21	−1.70	0.004	Crucian carp (*C. auratus*)	Unknown	This study
novel-miR-25	−1.53	1.07e−11	Crucian carp (*C. auratus*)	Unknown	This study

**Figure 6:**
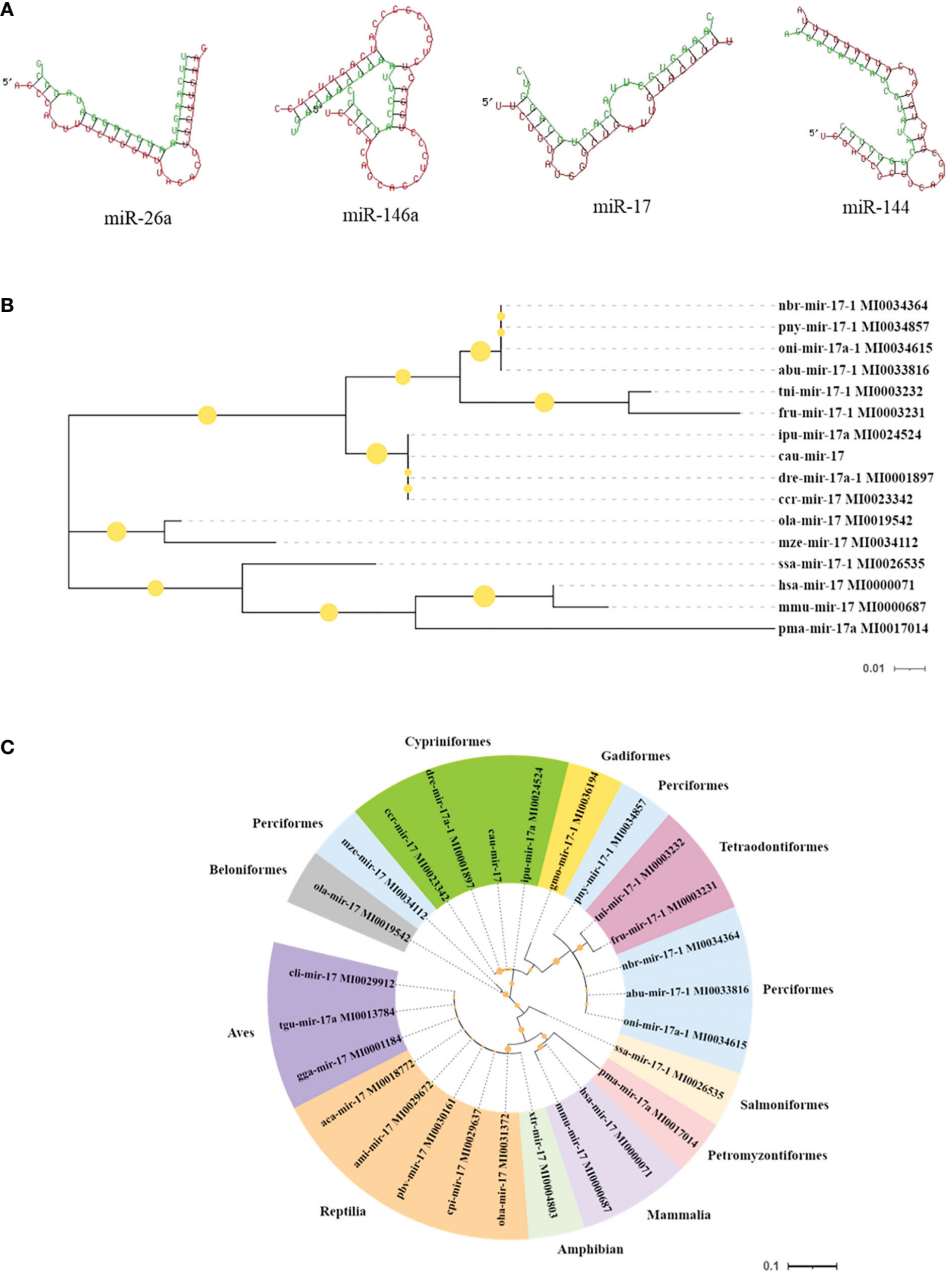
Molecular characteristics of the microRNAs (miRNAs) identified in the gills. **(A)** Predicted target sites of miR-17, miR-144, miR-146a, and miR-26a. **(B)** Species genetic relationship of miR-17 sequences. **(C)** Phylogenetic tree analysis of pre-miR-17 from the 26 species.

Moreover, the results of the key miRNA–mRNA analysis based on the relationship between negative expression and target gene prediction showed that miR-17, miR-26a, miR-144, and miR-146a bound to the 3′-UTR of the target genes *NF-κB*, *TLR4*, *TAB1*, and *IRAK1*, respectively (**Figure 6A**). Based on the evaluation of the multi-sequence alignment lineal homology for miRNAs, the miR-17 precursor sequence was comprehensively analyzed, which showed that the miR-17 precursor sequence of *C. auratus* had high similarity to the other 14 fish species, which is highly conserved in teleosts (**Supplementary Figure S1**). The phylogenetic tree results also showed that the miR-17 of *C. auratus* was closely clustered into one group with the zebrafish *D. rerio*, the common carp *C. carpio*, and the channel catfish *Ictalurus punctatus* (**Figure 6B**). To further analyze the evolutionary relationship of miR-17, we constructed MLE phylogenetic trees for the pre-miR-17 from 26 species, and similar results were observed for the evolutionary relationship in Cypriniformes (**Figure 6C**). In the miR-17 family, cau-miR-17 was very similar to dre-miR-17a-1, ccr-miR-17, and ipu-miR-17a, which were classified into the same branch within the phylogenetic tree. This revealed the sequence conservation of the miRNA precursor sequences of the same family among the different species of Cyprinid fish.

### GO and KEGG enrichment analyses of the target genes and proteins

**Figure 7:**
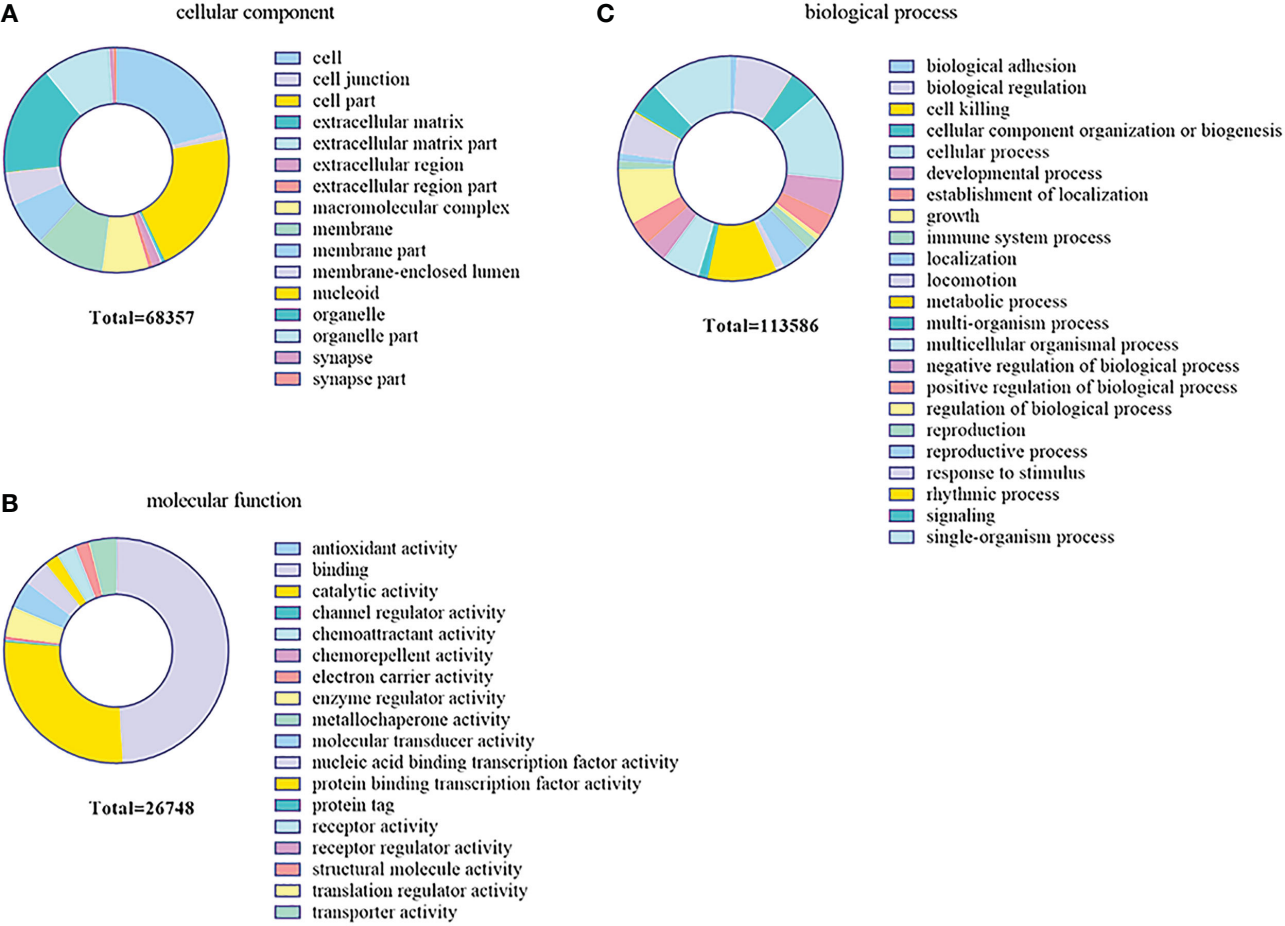
Gene Ontology (GO) enrichment analysis of the target genes predicted for the differentially expressed miRNAs (DEMs) in the gills. **(A)** Cellular component. **(B)** Biological process. **(C)** Molecular function.

In order to further investigate the function of the DEMs in the gill immune response of *C. auratus*, GO and KEGG enrichment analyses were performed to predict potential target genes and signaling pathways. A total of 104,211 putative target genes and 539 known miRNAs were screened in the gills. In addition, 17 unknown miRNAs and 45,326 target gene sequences were annotated in GO. These were mainly involved in biological regulation, cellular processes, metabolic processes, and biological adhesion processes. In the biological process GO terms, most of the genes were related to the cellular process, while the single-organism process, metabolic process, biological regulation, rhythmic process, and cell killing were lower. Regarding the cellular components, most genes were related to cells and cellular components, followed by organelle and macromolecular complex. In terms of molecular function, binding involved the most, followed by catalytic activity, enzyme regulator activity, molecular transducer activity, and nucleic acid binding transcription factor activity (**Figure 7**).

**Figure 8:**
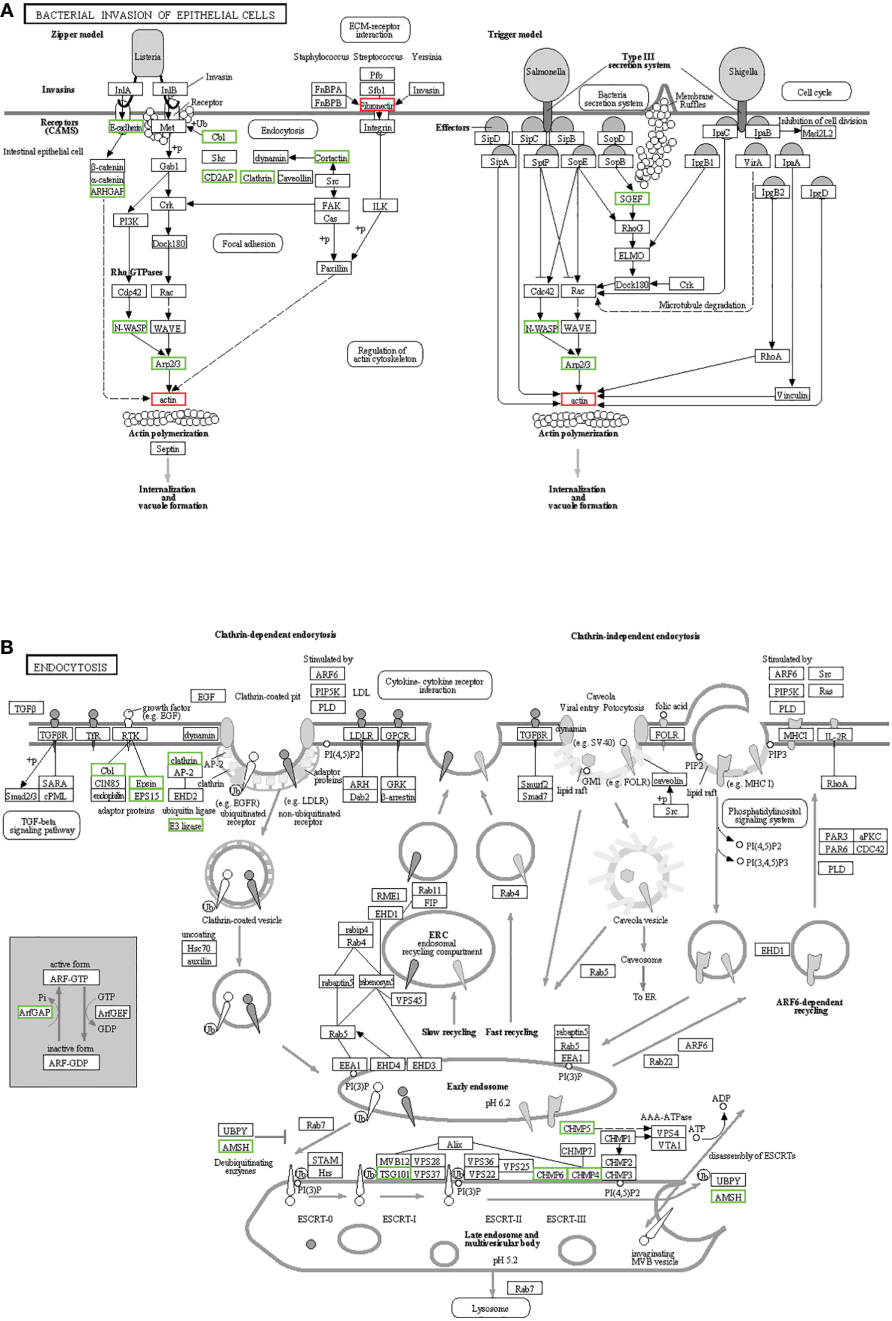
Kyoto Encyclopedia of Genes and Genomes (KEGG) pathways for the negative association of the identified proteins in the gills. **(A)** Bacterial invasion of epithelial cells. **(B)** Endocytosis. The differentially expressed proteins (DEPs) of the different pathways were upregulated (*red*) and downregulated (*green*).

KEGG enrichment analysis mainly focused on 306 signaling pathways, of which 33 were related to gill immunity, including metabolic pathways, cell adhesion molecules (CAMs), TLRs, NOD-like receptors (NLRs), natural killer cell-mediated cytotoxicity (NKCC), RIG-I-like receptor (RLRs), MAPK, Janus kinase signal transducer and activator of transcription (JAK-STAT), NF-κB, p53, Fc gamma receptor (FcγR)-mediated phagocytosis, T-cell receptor (TCR), and B-cell receptor (BCR) signaling pathways (**Table 3**). At the protein level, in order to identify the biological pathways that play key roles in the response to *A. hydrophila* infection, we performed iTRAQ and KEGG analyses of the DEPs. The significantly enriched immune pathways found included bacterial invasion of epithelial cells (14), complement and coagulation cascades (15), regulation of actin cytoskeleton (14), focal adhesion (8), antigen processing and presentation (10), FcγR-mediated phagocytosis (5), phagosome (14), pathogenic *Escherichia coli* infection (19), endocytosis (11), NF-κB and MAPK (6), and insulin signaling pathway (5) of DEPs (**Supplementary Table S5**). Notably, the network pathways for the association of the identified DEPs in the bacterial invasion of epithelial cells and endocytosis were significantly downregulated at the protein level in the gills (**Figure 8**).

**Table 3:** Immune-related Kyoto Encyclopedia of Genes and Genomes (KEGG) pathways enriched by the target genes in the gills of *Carassius auratus* challenged with *Aeromonas hydrophila*.

Pathway	Target genes^a^	Pathway ID
Metabolic pathways	4,123 (10.49%)	ko01100
Pathways in cancer	2,007 (5.11%)	ko05200
Regulation of actin cytoskeleton	1,746 (4.44%)	ko04810
Focal adhesion	1,635 (4.16%)	ko04510
MAPK signaling pathway	1,337 (3.4%)	ko04010
Chemokine signaling pathway	1,071 (2.73%)	ko04062
RNA transport	939 (2.39%)	ko03013
Phagosome	930 (2.37%)	ko04145
Biosynthesis of secondary metabolites	908 (2.31%)	ko01110
*Salmonella* infection	881 (2.24%)	ko05132
Fc gamma R-mediated phagocytosis	860 (2.19%)	ko04666
Ubiquitin-mediated proteolysis	813 (2.07%)	ko04120
Leukocyte transendothelial migration	806 (2.05%)	ko04670
Cell adhesion molecules (CAMs)	784 (1.99%)	ko04514
Cytokine–cytokine receptor interaction	784 (1.99%)	ko04060
Calcium signaling pathway	766 (1.95%)	ko04020
T-cell receptor signaling pathway	733 (1.87%)	ko04660
Natural killer cell-mediated cytotoxicity	688 (1.75%)	ko04650
Cell cycle	687 (1.75%)	ko04110
NOD-like receptor signaling pathway	679 (1.73%)	ko04621
NF-kappa B signaling pathway	675 (1.72%)	ko04064
JAK-STAT signaling pathway	661 (1.68%)	ko04630
B-cell receptor signaling pathway	563 (1.43%)	ko04662
*Vibrio cholerae* infection	523 (1.33%)	ko05110
Fc epsilon RI signaling pathway	472 (1.2%)	ko04664
Toll-like receptor signaling pathway	465 (1.18%)	ko04620
Apoptosis	450 (1.14%)	ko04210
Antigen processing and presentation	430 (1.09%)	ko04612
p53 signaling pathway	391 (0.99%)	ko04115
mTOR signaling pathway	368 (0.94%)	ko04150
RIG-I-like receptor signaling pathway	348 (0.89%)	ko04622
PPAR signaling pathway	298 (0.76%)	ko03320
Complement and coagulation cascades	255 (0.65%)	ko04610

*Target genes indicated with pathway annotation (39302).

### Spatiotemporal expression and regulation analysis of the key miRNAs/target genes

**Figure 9:**
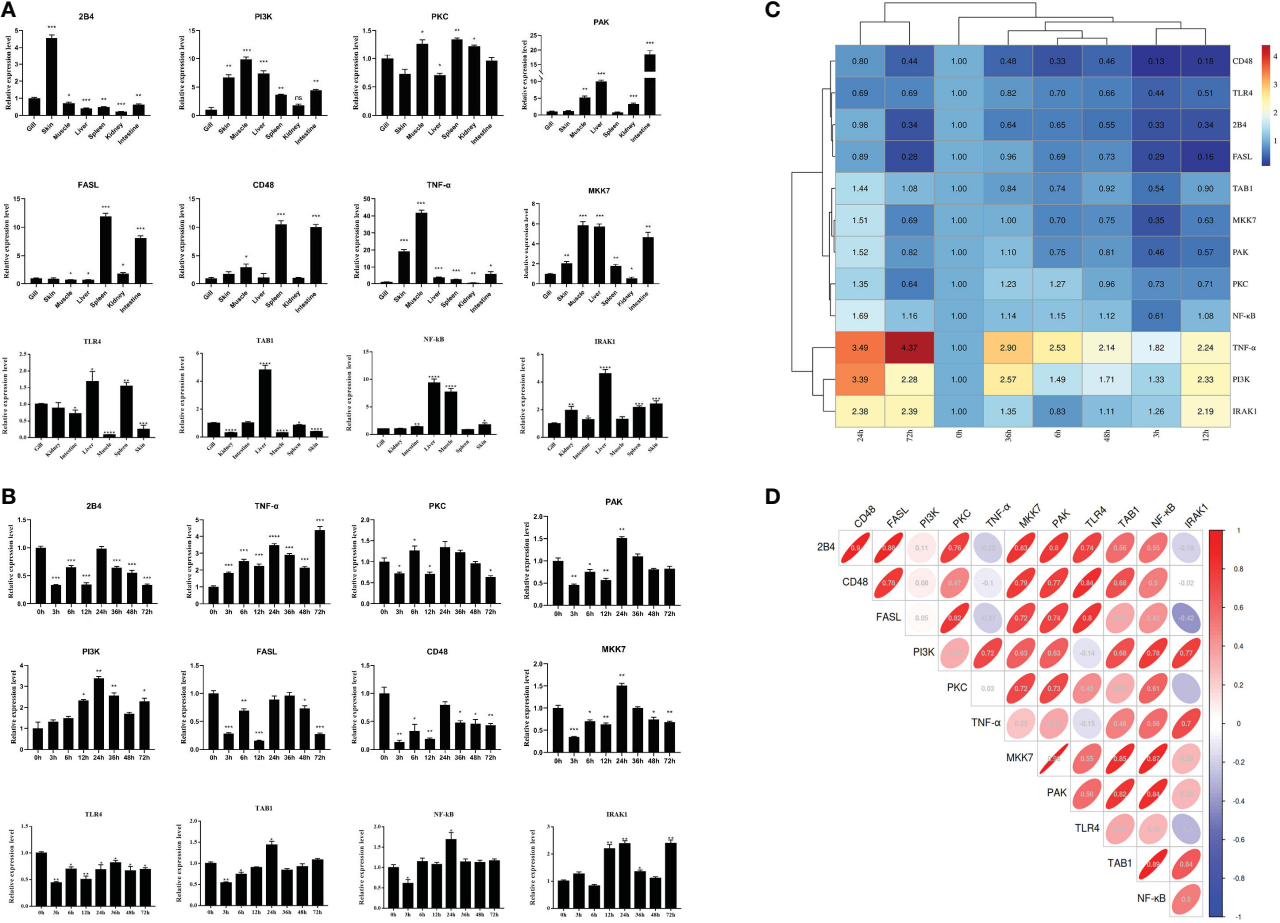
Expression changes of the immune-related target genes within the Toll-like receptor (TLR), mitogen-activated protein kinase (MAPK), and nuclear factor kappa B (NF-κB) inflammatory pathways of *Carassius auratus*. **(A)** Tissue distribution of the 12 microRNA (miRNA)-mediated potential target genes. **(B)** Temporal expression changes of the target genes in the gills. **(C)** Heat map analysis of the relative expression. **(D)** Correlation analysis of the selected target genes. Toll-like receptor 4 (*TLR4*), interleukin-1 receptor-associated kinase 1 (*IRAK1*), TGF-beta-activated kinase 1 and MAP3K7-binding protein 1 (*TAB1*), protein kinase C lambda (*PKC*), Fas ligand (*FASL*), nuclear factor kappa-B kinase subunit alpha-like (*NF-κB*), P21-activated kinase (*PAK*), phosphatidylinositol 3-kinase (*PI3K*), MAP kinase kinase 7 (*MKK7*), CD48 molecule (*CD48*), CD244 molecule (*2B4*), and tumor necrosis factor-α (*TNF-α*), which were determined by quantitative real-time PCR (qRT-PCR) assay. All data were from three independent triplicate experiments (*p < 0.05, **p < 0.01, ***p < 0.001, ****p < 0.0001).

**Figure 10:**
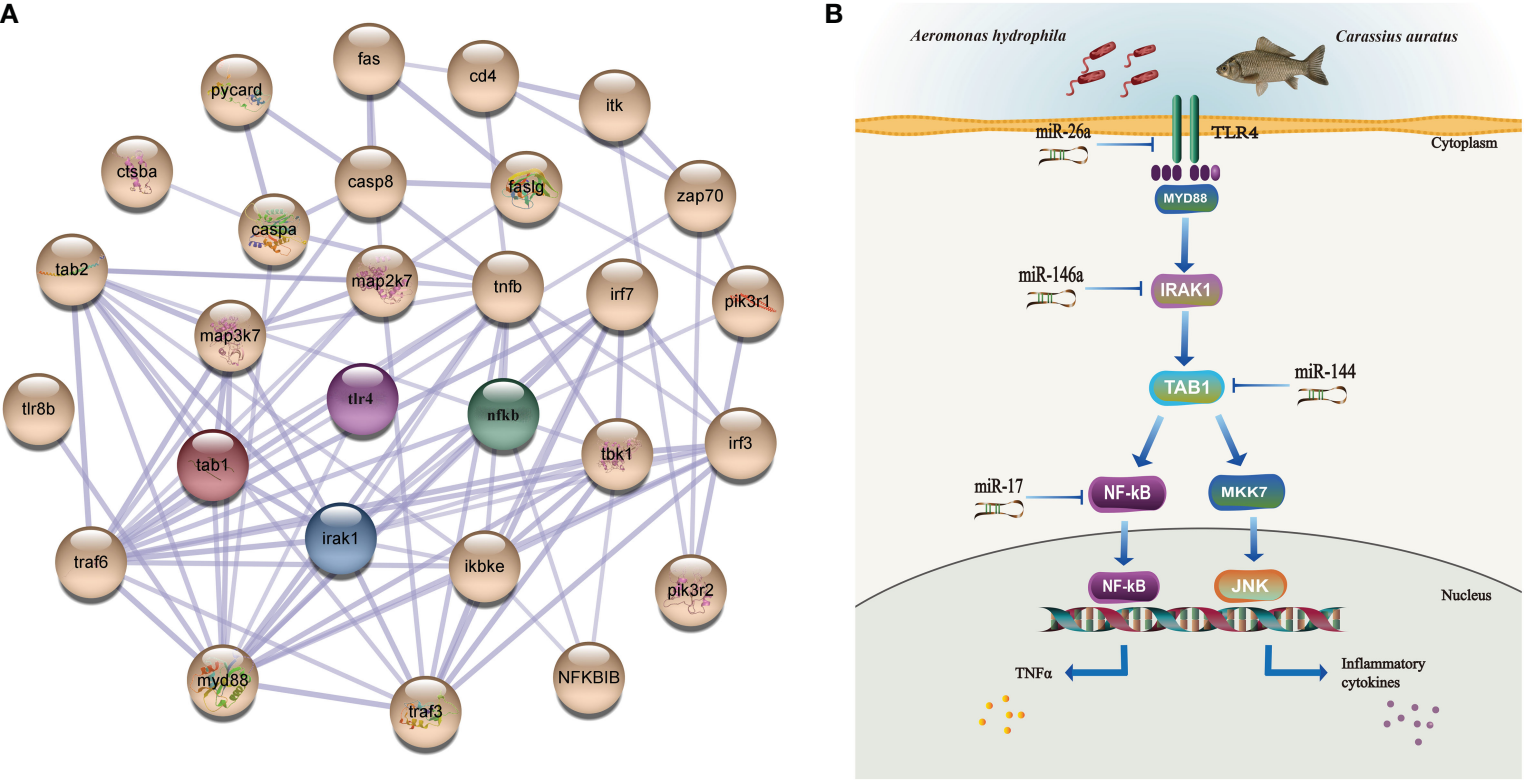
Interaction network analysis and illustration of the regulation mechanisms of the key miRNAs/target genes in the gills of *Carassius auratus* infected with *Aeromonas hydrophila*. **(A)** Interaction analysis of the protein–protein interaction (PPI) networks. *Different colored balls* represent the differentially expressed proteins (DEPs), the *node* indicates representative proteins of the corresponding pathway, and the *line thickness* denotes the strength of the predicted functional interactions. **(B)** Illustration of the regulation mechanism of the key miRNAs/target genes in the gills. miR-26a, miR-146a, miR-144, and miR-17 were identified in the gills, which were involved in the local immune regulation of the Toll-like receptor (TLR), mitogen-activated protein kinase (MAPK), and nuclear factor kappa B (NF-κB) signaling pathways by targeting Toll-like receptor 4 (*TLR4*), interleukin-1 receptor-associated kinase 1 (*IRAK1*), TGF-beta-activated kinase 1 and MAP3K7-binding protein 1 (*TAB1*), and nuclear factor kappa-B kinase subunit alpha-like (*NF-κB*).

The tissue distribution analysis of the miRNA-mediated target genes (e.g., *TLR4*, *TAB1*, *NF-κB*, *IRAK1*, *PKC*, *FASL*, and *CD48*) showed that the 12 immune-related genes exhibited tissue-specific expression and presented different expression levels in the examined tissues (**Figure 9A**). Furthermore, temporal expression analysis of the miRNA primary target genes within the TLR, NKCC, MAPK, and NF-κB signaling pathways showed that several of them, including *TLR4*, *2B4*, *FASL*, *CD48*, and *TAB1*, were mostly downregulated 72 h after *A. hydrophila* infection, with the expression levels of *TLR4*, *TAB1*, *CD48*, and *2B4* at 3 hpi at the lowest points. On the other hand, the expression levels of *NF-κB* and *IRAK1* were upregulated during the challenge, with the highest expressions at 24 and 72 hpi being 1.69 and 2.39 fold change, respectively. The expression levels of *PKC*, *MKK7*, and *PAK* in the gills showed a trend of early decline, middle rise, and late decline, in which the lowest expression was shown by *MKK7* and *PAK* at 3 hpi and the highest expression of the three genes was at 24 hpi. Furthermore, *TNF-α* and *PI3K* showed an upward trend within 72 hpi. The expression of *TNF-α* was the highest at 72 hpi, that of *PI3K* was 3.39-fold at 24 hpi, and that of *FASL* was at 12 hpi in the gills of *C. auratus* (**Figure 9B**). It is worth noting that the expression characteristics of the *PI3K* gene were positively associated with those of *TAB1*, *NF-κB*, and *IRAK1* (**Figures 9C, D**, **Supplementary Figure S2**). The qRT-PCR data of the detected candidate genes exhibited similar expression trends to the proteins in the iTRAQ analysis, which showed a significant correlation (Pearson’s correlation coefficient of 0.70, *p* < 0.01). The protein–protein interaction (PPI) analysis revealed that the potential key miRNA target proteins (e.g., TLR4, IRAK1, TAB1, NF-κB, MyD88, TRAF3/6, MAPK, IRF3/7, FASL, TNF, and CD4) showed significant correlation in the interaction network of the infected gills of *C. auratus* (**Figure 10A**). Thus, it was speculated that the molecular mechanism of the gill immune response in *C. auratus* against *A. hydrophila* infection might be the regulation of the production of cytokines and inhibition of excessive inflammatory response. Further insight into the regulation mechanism of the key miRNAs (i.e., miR-26a, miR-146a, miR-144, and miR-17), which were involved in the gill local immune regulation of the TLR, MAPK, and NF-κB signaling pathways, was also drawn (**Figure 10B**).

## Discussion

With the development of intensive aquaculture in China, the outbreak of bacterial diseases of the crucian carp *C. auratus* has become a serious problem, causing economic losses (18, 27). Recently, the spotlight on miRNA biomarkers, target genes, and immune regulation has been of great significance for the prevention and treatment of diseases in teleost fish (5, 7, 8). Mucosal immunity (i.e., gills, skin, and intestine) is the host’s first line of defense against pathogen infection (3, 18, 27), which involves different signaling pathways that are regulated by complex mechanisms; however, most of the miRNAs are still unclear in fish (8, 9). As essential regulatory factors of gene expression in the biological process, miRNAs play important roles in cell differentiation, organ development, and immune progression (9, 44). Previous studies on the miRNAs of teleosts showed that they are pivotal regulators of the inflammatory response in teleosts (45–47), indicating that they are involved in the regulation of early development, organogenesis, cell differentiation and homeostasis, growth, reproduction, and immunity (48, 49). Characterization of the miRNAs and their target genes in the internal organs (e.g., kidney, spleen, and liver) and skin of *C. auratus* was subsequently performed using deep sequencing analysis (5, 18). The miRNA expression and its regulatory mechanism in the gills remain unclear in *C. auratus*. The present study used a high-throughput sRNA transcriptome sequencing technique to construct an sRNA library from the gills of *C. auratus* infected with *A. hydrophila* based on the Illumina HiSeq 2000 platform. The results of this study provide a scientific basis for the elucidation of the miRNAs and target genes involved in the molecular mechanisms in the gill mucosal immune response of teleost fish.

Current evidence in fish has demonstrated miRNAs to be key regulators that play essential roles in immune response and negatively regulate the expression of genes at the posttranscriptional level (47, 50). In the crucian carp *C. auratus*, characterization of the conserved and novel miRNAs (e.g., miR-10, miR-17, and miR-122) has been recently performed using deep sequencing and prediction of miRNA targets (5). Previous studies demonstrated that the significant upregulation of miR-21, miR-146a, and miR-200a indicates their involvement in the process of skin mucosal immune response in *C. auratus* infected with *A. hydrophilic* (18). miRNAs (e.g., miR-21, miR-145, and miR-146a) are also involved in the inflammatory and immune responses to the miiuy croaker *M. miiuy* and the zebrafish *D. rerio* (11, 51), and several miRNAs (e.g., miR-21, miR-122, and miR-192-5p) were found to be differentially expressed in the *Vibrio anguillarum*-challenged *M. miiuy* (14, 15). Recent miRNA expression analysis of *D. rerio* infected with *Vibrio parahaemolyticus* has indicated that 37 known miRNAs (e.g., dre-miR-141-5p, dre-miR-200a-5p, and dre-miR-192) were differentially expressed, which regulate signal conduction, hematopoiesis, and protein synthesis (52). The miRNA genes in the Atlantic cod *Gadus morhua* determined by qRT-PCR analysis showed that several miRNAs (e.g., miR-144, miR-26a, and miR-200a) were overexpressed and were mainly involved in the regulation of growth, metabolism, and immune response (48). In this study on the gills of *C. auratus* challenged with *A. hydrophila*, a total of 1,148 known and 17 unknown miRNAs were obtained using the high-throughput Illumina HiSeq 2000 sequencing platform, with some of the miRNAs (e.g., miR-10, miR-106a, miR-146b-3p, miR-202-5p, miR-17, and miR-145) found to be significantly differentially expressed. Notably, the expression levels of miR-144, miR-145, and miR-146a were significantly upregulated, while those of miR-17, miR-21, miR-26a, miR-122, and miR-200a were downregulated, thereby indicating that they could serve the function of regulating the expression of the target genes in the gill mucosal immune response. Furthermore, the phylogenetic analysis revealed that the miR-17 of *C. auratus* showed a highly conservative evolution in teleost; however, some of the known miRNAs (e.g., miR-10, miR-145, and miR-155) and several novel miRNAs (e.g., novel-miR-4, novel-miR-10, and novel-miR-12), which may have resulted from differences in the species specificity of teleosts, still require further research.

Fish innate immunity is the first line of host defense and mainly comprises pattern recognition receptor signaling pathways (13, 53). The recently identified miRNAs play important roles in the regulation of the inflammatory and immune responses, indicating a miRNA-mediated TLR/NF-κB signaling pathway (54, 55). In teleost fish, it is worth noting that the miRNAs were significantly related to immune defense, such as the NLR, apoptosis, JAK-STAT, and MAPK singling pathways that are involved in various biological processes (8, 56), consistent with the results of this study on *C. auratus*. Recent research has found that miR-7a participates in PI3K regulation (17), and miR-145-5p and miR-122 have been proven to regulate the RLR signaling pathway (38, 57). We previously reported on miRNAs in the skin of *C. auratus* infected with *A. hydrophila*, indicating that the skin immune response involved the TLR, MAPK, JAK-STAT, and phagosome pathways (27). The present study also found that a lot of the miRNAs and their target genes (i.e., *TLR4*, *TAB1*, *IRAK1*, and *NF-κB*) in the gills of *C. auratus* infected with *A. hydrophila* were focused on inflammatory and immune responses, such as the TLR, MAPK, and NF-κB inflammatory pathways. Thereby, it was suggested that these key miRNAs and their target genes may be involved in the immunomodulatory process in the gill mucosal signaling pathways, promoting immune response and protecting the organism against overwhelming inflammation after bacterial infection. However, the roles of the miRNAs in teleost gills in relation to pathogens still need to be further studied in the future.

In addition, the miRNAs interacting with the target genes and proteins were not only associated with immune response (7, 45) but also played important roles in regulating the regulators of the immune network (28, 58). In *M. miiuy* infected with *Vibrio harveyi*, miR-148 targeted and negatively regulated the expression of the *MyD88* gene, and overexpression of miR-148 inhibited the inflammatory cytokine production of both *IL-6* and *IL-1β* (16). miR-21 also upregulated and targeted the gene *IRAK4* (11), thereby preventing excessive inflammation response. In *M. miiuy* infected with *V. anguillarum*, miR-200a-3p was shown to be involved in the modulation of *TLR1* expression (57). In the gills of *D. rerio* after *A. hydrophila* and *Edwardsiella piscicida* infection (12), miR-146a suppressed the expression of the immune-related genes and was involved in the regulation of excess inflammatory responses (e.g., *TLR4*, *IL-1β*, and *TNF-α*), and it also promoted viral replication by targeting *TRAF6* in the orange-spotted grouper *Epinephelus coioides* (59). In vertebrates, several studies verified the involvement of *NOD1* in the regulation of the antibacterial immune response (60, 61), while miR-144 and miR-217-5p targeted and sequentially inhibited the gene expression of *NOD1* (13). Studies on the liver of zebrafish indicated that miR-731 served the function of directly targeting *DKK3b*, while several miRNAs (e.g., miR-200b-5p, miR-146b, and miR-731) and their target genes (i.e., *IL10*, *IRAK1*, and *TRAF6*) regulated the immune response in the spleen after *Streptococcus parauberis* infection (24, 62). The regulation of NF-κB revealed that miR-144 negatively regulated signaling transduction by targeting the *IL-1κ* gene (49). Moreover, several miRNAs (e.g., miR-122, miR-192, and miR-148) have been identified for the regulation of the target genes that participate in innate immunity (14–16). In this study on the gills of *C. auratus* infected with *A. hydrophila*, we found four miRNAs (e.g., miR-26a, miR-144, miR-146a, and miR-17) that were significantly differentially expressed in the gills, which might regulate their target genes involved in the TLR, MAPK, and NF-κB inflammatory pathways. These results present new ideas for further studies of the miRNA regulatory mechanism in the gill immune response of carp against infections.

## Conclusion

To our knowledge, this is the first study on the multi-omics analysis of the miRNAs and the protein profiles in the gills of the crucian carp *C. auratus* after *A. hydrophila* infection. Based on conventional histopathological and immunohistochemical studies, the expression profiling and target gene analysis revealed that the miRNAs were significantly differentially expressed in the gills, thereby indicating that the key miRNAs (i.e., miR-17, miR-26a, miR-144, and miR-146a) play crucial roles in the local immune response of *C. auratus* against bacterial infection. The target genes of the miRNAs involved in multiple signaling pathways mainly included TLR, MAPK, and NF-κB inflammatory responses. This study provides a detailed description of the mechanism of the regulation of miRNAs in the gills and contributes to the understanding of the miRNA-mediated local immune response in Cyprinidae fish.

## Data availability statement

The data presented in the study are deposited in the NCBI sequence read archive (SRA) repository, accession number: PRJNA923102.

## Ethics statement

The animal study was reviewed and approved by the Tianjin Agricultural University Institutional Animal Care and Use Committee (TJAU-IACUC).

## Author contributions

AL conceived the project. XH and AL provided the experimental instruments, reagents, and animals. JH and JB conducted the animal experiments. JH analyzed the data and wrote the manuscript. AL revised the manuscript. All authors contributed to the article and approved the submitted version.

## Supplementary material

Supplementary Figure 1 The multiple sequences alignments of precursor miR-17 sequences.

Supplementary Figure 2 Cloning analysis of target-gene PI3K in *C. auratus*. **(A)** PCR amplification electrophoresis and positive clone detection. **(B)** Prediction of PI3K secondary structure. **(C)** Prediction of PI3K tertiary structure. **(D)** Phylogenetic tree of PI3K amino acid sequence. M is DNA standard DL 2000 bp; lane 1 is PI3K-1, lane 9 is PI3K-2, lanes 2-8 are pMD18-T PI3K-1 positive clones, lanes 10-13 are pMD18-T PI3K-2 positive clones.

Supplementary Table 1 Selected miR-17 sequences for 26 different species from the miRBase database.

Supplementary Table 2 Primers for qRT-PCR method detection of crucian carp *Carassius auratus.*

Supplementary Table 3 Summary of high-throughput sequencing from gill libraries of *C. auratus* infected with *A. hydrophila*.

Supplementary Table 4 Summary of sequenced miRNAs in the gills of *C. auratus.*

Supplementary Table 5 The iTRAQ analysis of enriched KEGG pathways in the gills of *C. auratus* challenged with *A. hydrophila*.
