# Supplementary figures and images for: Multiomics analysis revealed miRNAs as potential regulators of the immune response in Carassius auratus gills to Aeromonas hydrophila infection
Source: Front Immunol. 2023 Feb 3;14:1098455. doi: 10.3389/fimmu.2023.1098455 (PMC9938762; doi:10.3389/fimmu.2023.1098455)

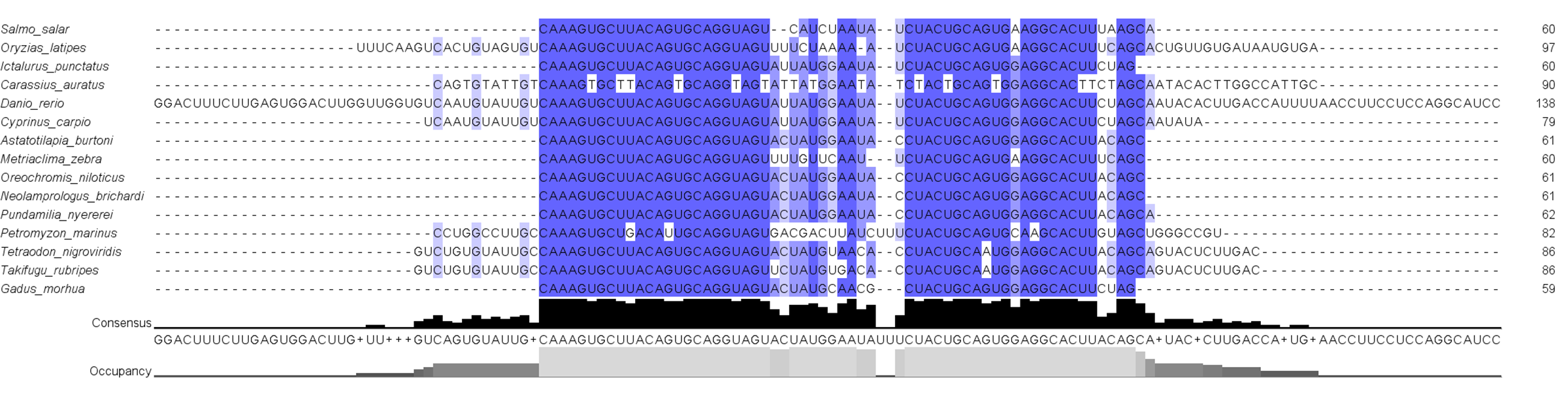

Supplement: Supplementary Figure 1 — The multiple sequences alignments of precursor miR-17 sequences. [file Image_1.tif]

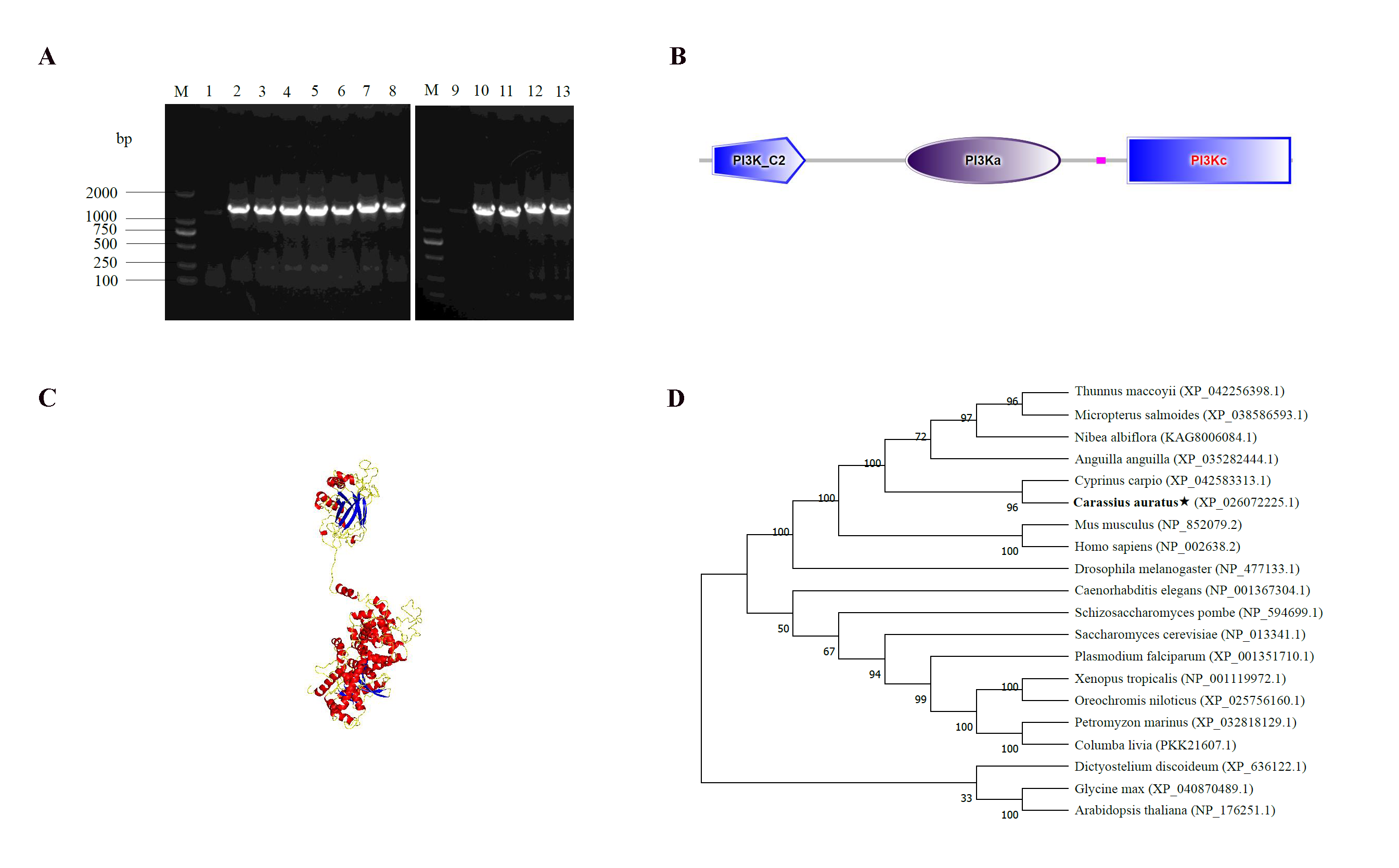

Supplement: Supplementary Figure 2 — Cloning analysis of target-gene PI3K in C. auratus. (A) PCR amplification electrophoresis and positive clone detection. (B) Prediction of PI3K secondary structure. (C) Prediction of PI3K tertiary structure. (D) Phylogenetic tree of PI3K amino acid sequence. M is DNA standard DL 2000 bp; lane 1 is PI3K-1, lane 9 is PI3K-2, lanes 2-8 are pMD18-T PI3K-1 positive clones, lanes 10-13 are pMD18-T PI3K-2 positive clones. [file Image_2.tif]
